# Ligand Binding Stabilizes Cellulosomal Cohesins as Revealed by AFM-based Single-Molecule Force Spectroscopy

**DOI:** 10.1038/s41598-018-27085-x

**Published:** 2018-06-25

**Authors:** Tobias Verdorfer, Hermann E. Gaub

**Affiliations:** Lehrstuhl für Angewandte Physik and Center for Nanoscience, Ludwig-Maximilians-Universität, 80799 Munich, Germany

## Abstract

The cohesin-dockerin receptor-ligand family is the key element in the formation of multi-enzyme lignocellulose-digesting extracellular complexes called cellulosomes. Changes in a receptor protein upon binding of a ligand - commonly referred to as allostery - are not just essential for signalling, but may also alter the overall mechanical stability of a protein receptor. Here, we measured the change in mechanical stability of a library of cohesin receptor domains upon binding of their dockerin ligands in a multiplexed atomic force microscopy-based single-molecule force spectroscopy experiment. A parallelized, cell-free protein expression and immobilization protocol enables rapid mechanical phenotyping of an entire library of constructs with a single cantilever and thus ensures high throughput and precision. Our results show that dockerin binding increases the mechanical stability of every probed cohesin independently of its original folding strength. Furthermore, our results indicate that certain cohesins undergo a transition from a multitude of different folds or unfolding pathways to a single stable fold upon binding their ligand.

## Introduction

Cellulosomes are extracellular multi-enzyme complexes produced by many microorganisms for the efficient degradation of cellulose - nature’s most abundant biopolymer. This degradation of plant cell-wall polysaccharides is accomplished *via* the spatial organization of a variety of cellulolytic enzymes through scaffolding proteins (i.e. scaffoldins) and boosted by its synergistic effects^1,2^. This complex network formation is driven by non-covalent, high-affinity receptor-ligand protein domains called cohesins (Coh) - which comprise the majority of scaffoldins - and dockerins (Doc) - which are typically connected to enzymatic domains^3^. The Coh-Doc complex has become a popular model system to study different aspects of protein-protein interactions, such as receptor-ligand binding specificities, affinities and strengths, dual binding conformations^4–8^, and the mechanical stability of cohesin domains - and thus the integrity of the cellulosomal scaffoldins themselves. In particular, it was shown that bridging cohesins, which are subjected to mechanical stress when the cell is anchored to its substrate, are able to withstand higher forces than hanging cohesins in order to remain folded and thus functional^9–11^. While these studies focussed exclusively on cohesins from the scaffoldin CipA of *Clostridium thermocellum* and the scaffoldin ScaA of *Acetivibrio cellulolyticus* in isolation, we now investigate the impact of dockerin binding on the mechanostability of cohesin folds.

The binding of a ligand can often change the fold and thereby the function of a receptor protein. For example, G protein-coupled receptors exhibit a wide variety of signaling behaviours in response to different ligands^12^. Ligand binding can also reduce the receptor’s conformational folding space, as in the case of many intrinsically disordered proteins that only undergo folding upon binding to their physiological partner^13^. Furthermore, the binding of a protein ligand can result in significant improvement of a receptor’s mechanical stability without introducing major structural changes, as reported by Cao *et al.*^14^. In agreement with this previous study, we discovered here that all probed cohesin domains markedly increase their force resilience upon binding of their dockerin ligand. This finding was enabled by combining several recent developments in atomic force microscopy-based (AFM) single-molecule force spectroscopy (SMFS), including site-specific protein immobilization, molecular fingerprint domains, highly specific and strong pulling handles, and *in vitro* transcription/translation IVTT-based microarray-format sample preparation^10,15,16^. Taken together, these techniques allow for a high-throughput characterization of the mechanical stability of a library of receptors - without and with a ligand bound - in a single automated AFM-SMFS experiment.

**Figure 1:**
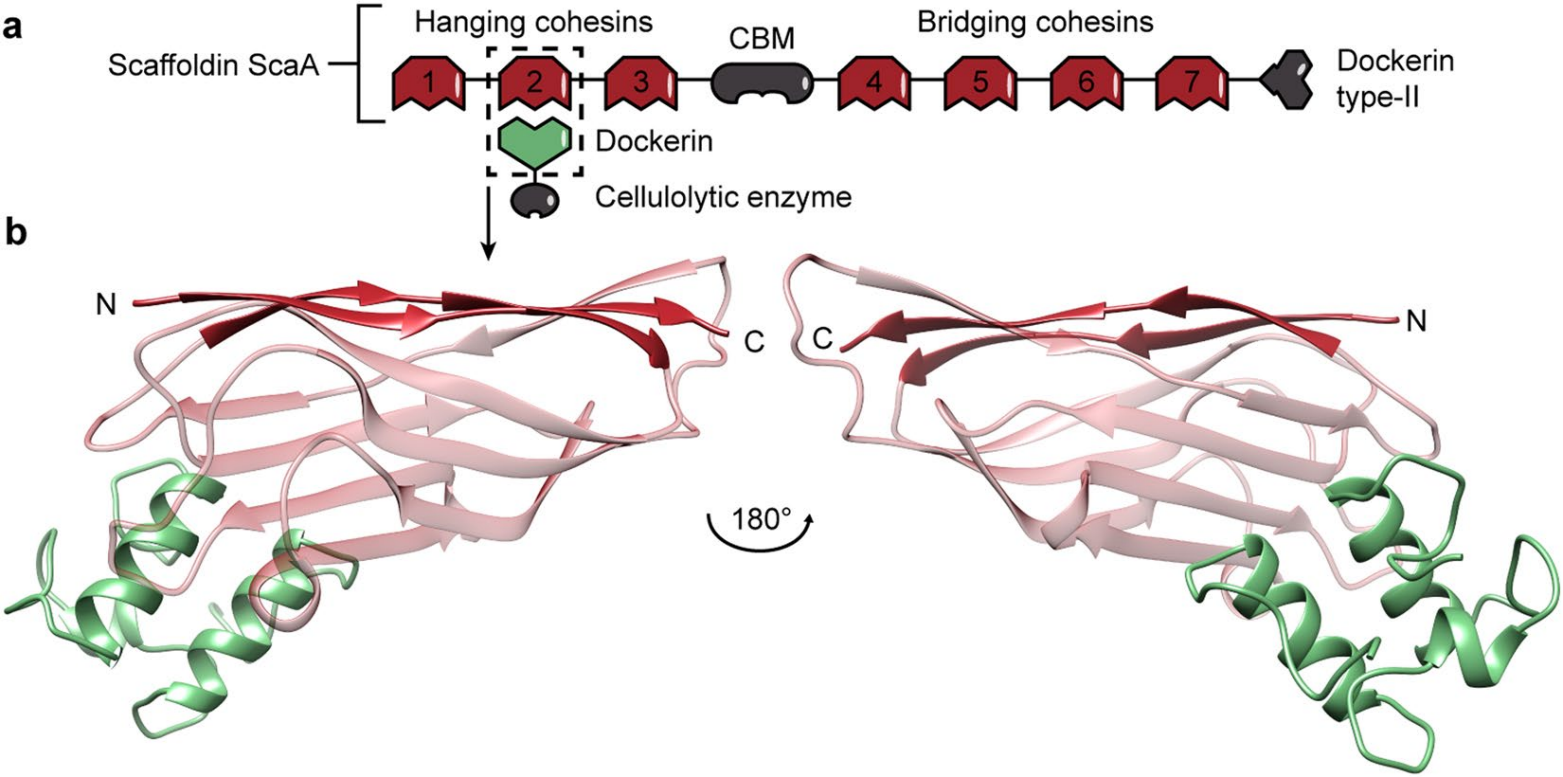
Schematic representation of scaffoldin ScaA and cohesin-dockerin binding geometry. (**a**) *A. cellulolyticus’* scaffoldin ScaA consists of seven type-I cohesins. Three mechanically weaker hanging cohesins (Coh1-Coh3) are located at the N-terminal end of the scaffoldin, while four mechanically stronger bridging cohesins (Coh4-Coh7) are located between a CBM and a type-II dockerin. The CBM and the type-II dockerin anchor the scaffoldin to the cellulolytic substrate and to the cell, respectively. The binding specificity of the cohesins is orthogonal to the type-II dockerin, thus they do not bind one another. Enzyme-bearing dockerins (green) can bind to each of the cohesins. ScaA’s N-terminal glycoside hydrolase is not shown for simplicity. (**b**) Molecular representation of a typical cohesin-dockerin pair (PDB: 4DH2), as highlighted in Fig. 1(a). The cohesin is shown in red and the dockerin in green. The cohesin’s binding interface is at the opposite site of its main structural element, the mechanical clamp motif, which consists of hydrogen bonds between parallel N- and C-terminal beta sheets.

Our study focuses on all seven cohesins from *A. cellulolyticus’* scaffoldin ScaA (Fig. 1(a)) and two cohesin mutants from our previous work^10^. Scaffoldin ScaA contains three major domains: a series of three hanging cohesins, a central carbohydrate-binding module (CBM) that anchors the cell to its substrate, and a series of four bridging cohesins. Bridging cohesins are mechanically stronger than hanging cohesins, although these two classes both bind *A. cellulolyticus’* type-I dockerins^17^. Additionally, we tested two mutants of ScaA’s mechanically weakest cohesin Coh1 from our previous work, where we identified minimal mutations that significantly increased the mechanical stability of Coh1. Mutant T107S displayed an atypical bimodal unfolding force distribution and we hypothesized that either strongly differing fold conformations or unfolding pathways could cause such a behaviour. Mutant T107S was included in this study to clarify if it can bind dockerins and whether binding of a dockerin would stabilize its fold or alter its unfolding behaviour. Furthermore, we included mutant GGS, which is a triple mutant of Coh1 with the mutations A105G, P106G, and T107S. This mutant showed the largest increase in mechanical stability when compared to wild-type Coh1.

The high mechanical resilience of cellulosomal cohesins is predominantly attributed to the so-called mechanical clamp motif: a set of backbone hydrogen bonds formed between the N- and C-terminal β-strands of the cohesin protein domains (Fig. 1(b)). Previous studies that pulled cohesin from its termini - including all-atom steered molecular dynamics simulations - have identified the mechanical clamp motif as their main structural element^9,10^. Since the cohesin’s dockerin binding interface is located at the opposite side of the protein, a mechanically stabilizing effect of dockerin binding would thus have to be propagated through the cohesin fold to affect its unfolding energy landscape.

## Results and Discussion

### Experimental setup

**Figure 2:**
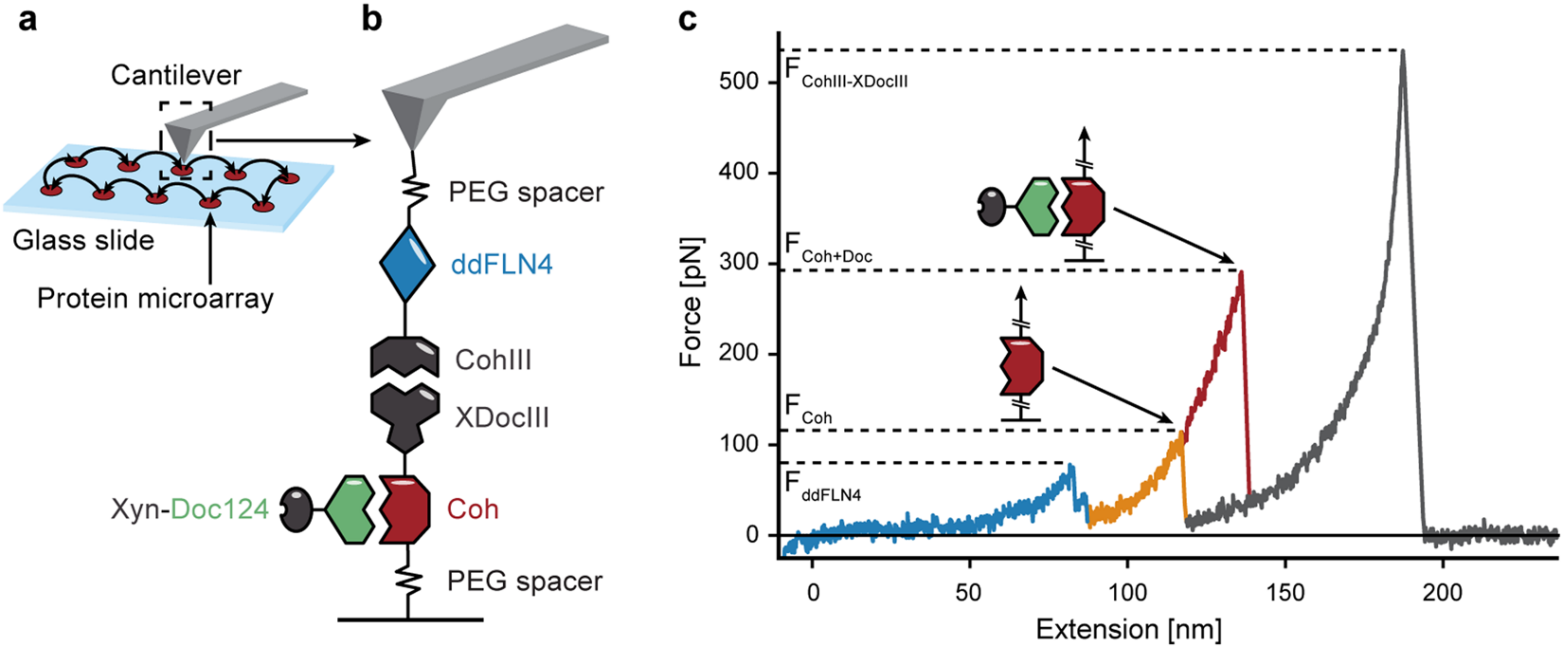
Experimental design and exemplary force curve. (**a**) One AFM cantilever can probe a library of different protein constructs, which are immobilized on a glass slide in a microarray format. This protocol allows for high experimental throughput and precise relative force comparability. (**b**) A fusion protein consisting of a ddFLN4 fingerprint domain and a CohIII is immobilized on the cantilever. Fusion proteins, each consisting of a cohesin of interest (Coh) as well as an XDocIII, are expressed and immobilized in a microarray format on a glass slide. The CohIII-XDocIII receptor-ligand pair serves as a highly specific pulling handle. At a later stage in the experiment, Xyn-Doc124 is added to the experimental buffer solution and can then bind to each Coh. (b) Overlay of two exemplary force-distance curves when retracting the cantilever with constant speed. First, the poly-protein stretches and the ddFLN4 fingerprint domain unfolds in two steps and at a relatively low force F_FP_ (blue). The poly-protein is stretched further and the cohesin domain unfolds at F_Coh_ (orange). In the presence of Xyn-Doc124, the cohesin unfolds at a higher unfolding force F_Coh+Doc_ (red). Finally, the CohIII-XDocIII pulling handle unbinds at F_CohIII-XdocIII_ (grey) and the force ultimately drops back to zero.

A library of fusion proteins containing the cohesins of interest is expressed and immobilized at spatially separated positions in a microarray format on glass slide using a cell-free, one-pot protein expression and pulldown reaction^10^. In short, a silicone mask is placed on a glass slide to compartmentalize the surface into several microwells with a diameter of about 5 mm. All microwells are functionalized with reactive PEG-linkers and filled with a one-pot reaction mix consisting of the *in vitro* transcription/translation (IVTT) system, the plasmid DNA encoding for the fusion proteins, and the enzyme facilitating the site-specific protein pulldown (phosphopantetheinyl transferase). In a single incubation step, this mixture results in cell-free protein synthesis, and simultaneous covalent ligation of the proteins onto the surface. The spatially separated protein spots can be probed in series using a single functionalized cantilever (see Figure 2(a)). This parallelized sample preparation method thus allows for quick mechanical phenotyping of protein libraries through AFM-SMFS.

Figure 2(b) shows the molecular pulling configuration. We chose the type-III cohesin-dockerin complex (CohIII-XDocIII) from Ruminococcus flavefaciens as a pulling handle due to its high specificity, long-term stability and high unbinding force^6^. The CohIII-XDocIII interaction is not expected to bind the type-I cohesins and dockerins from A. cellulolyticus, since the interaction between cohesins and dockerins was shown to be highly type- and species-specific^17^. The fusion proteins immobilized in the individual microarray spots on the glass slide each consist of a cohesin of interest (labeled Coh in Figure 2(b)) and an XDocIII. On the cantilever side we immobilized CohIII-ddFLN4 fusion proteins. ddFLN4 is the 4th immunoglobulin rod filamin domain from Dictyostelium discoideum and serves as a molecular fingerprint^18,19^. Its molecular unfolding pattern (rupture force and contour length increment) is used to identify traces with single, specific interactions. As a binding partner for the cohesins under investigation, we chose the dockerin from A. cellulolyticus’ enzyme-bearing Cel124A, as it has been shown to bind ScaA’s cohesins^17^. Subsequently, we designed a xylanase-dockerin124 (Xyn-Doc124) fusion construct derived from it. After all cohesins are probed without a dockerin bound, Xyn-Doc124 was added to the measurement buffer. We used a concentration of 5 μM, which is well above the typical affinity constant of a type-I cohesin-dockerin pair of ~10-11 M, thus ensuring that the vast majority of cohesin domains should have a dockerin bound during measurement^4^.

With this experimental design, all cohesins of interest are probed using a single cantilever *via* the CohIII-XDocIII pulling handle in a single high-throughput experiment. The cantilever automatically cycles through the protein microarray, acquiring hundreds of force-distance traces for each Coh before moving to the next position. When the cantilever approaches the surface in any of the microarray protein spots, a CohIII-XDocIII receptor ligand interaction can form. The cantilever retracts with constant speed, stretching the polypeptide chain. A typical AFM SMFS force-distance trace is shown in Fig. 2(c). First, the ddFLN4 domain unfolds in a recognizable two-step pattern, followed by the unfolding of the cohesin domain and the final unbinding of the CohIII-XDocIII pulling handle. It is possible that the CohIII unfolds as the CohIII-XDocIII bond ruptures, but our constant yield of force curves over 24 hours and several thousand force traces is a clear indication that CohIII either stays intact during the course of the experiment or quickly refolds after unfolding. The rupture force of the individual peaks (F_ddFLN4_, F_Coh_ and F_CohIII-XdocIII_, respectively) is extracted from the data and analysed further. After collecting sufficient unfolding data of the bare cohesins, the dockerin is added to the experimental buffer and the AFM continuously probes the ligand-bound cohesins. When Xyn-Doc124 is present in the measurement buffer, a higher cohesin unfolding peak is typically recorded (F_Coh+Doc_ > F_Coh_).

By using a single cantilever through all conditions, calibration errors of up to ~15% that normally result from individual AFM-based SMFS measurements can be circumvented^20^. We therefore obtain comparable absolute force data of all probed cohesins both with and without the dockerin bound.

### Cohesin mechanostability increases upon binding of dockerin

**Figure 3:**
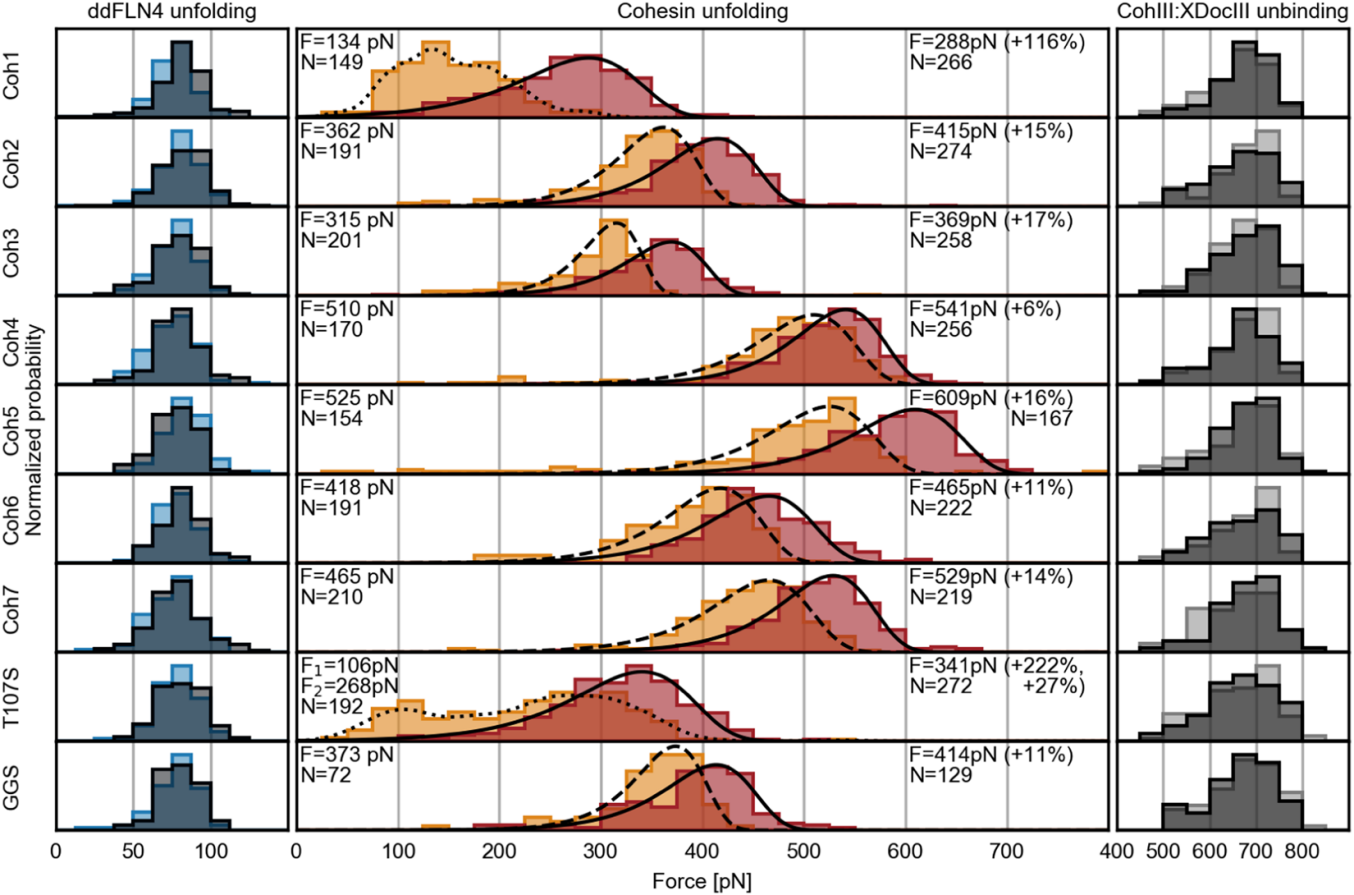
Unfolding and rupture force histograms without and with Xyn-Doc124 in solution. Both the unfolding force histograms of the ddFLN4 fingerprint (left column, bright and dark blue without and with dockerin in solution, respectively) and the unbinding force histograms of the CohIII-XDocIII pulling handle (right column, bright and dark grey without and with dockerin in solution, respectively) are independent of the cohesin under investigation and are unaffected by the presence or absence of Xyn-Doc124 during the measurement. All cohesins under investigation show their expected and previously known unfolding force distributions without Xyn-Doc124 in solution (middle column, orange)^10^. All but Coh1 and T107S can be fitted adequately using the Bell-Evans model to obtain their most probable rupture forces (dashed lines). The rupture force histograms of Coh1 and T107S were approximated using KDEs (dotted lines) to obtain the most probable rupture forces. In the presence of Xyn-Doc124, the unfolding force distributions of all cohesins are shifted towards higher forces (middle column, red) and can be fitted using the Bell-Evans model (solid lines). Forces F on the left and right side of the middle column represent the most probable rupture forces of the cohesins without and with Xyn-Doc124 in solution, respectively, and their relative change in percent. Numbers N on the left and on the right side of the middle column represent the number of cohesin unfolding force data points in the histograms without and with dockerin in solution, respectively. We found that changing the bin size within a range of 10 pN to 50 pN did not notably alter the fits of the Bell-Evans model to the cohesin unfolding force histograms or the obtained most probable unfolding forces. We therefore chose the same bin size for all constructs within a column of histograms (25 pN for all ddFLN4 and cohesin unfolding events, and 50 pN for all CohIII-XDocIII unbinding events), to provide good comparability by eye. All data were recorded using a single cantilever with a spring constant of 143 pN/nm at a retraction speed of 1600 nm/s in a 24-hour automated SMFS experiment. For more information see Table S1.

Figure 3 shows the unfolding force distributions of the ddFLN4 fingerprint domain (left column, blue) and of the cohesins under investigation (middle column, red), as well as the unbinding force distribution of the orthogonal CohIII-XDocIII pulling handle (right column, grey). Data collected without and with Xyn-Doc124 in solution are represented in dark and bright colors, respectively. The force histograms of both the ddFLN4 fingerprint and of the CohIII-XDocIII pulling handle agree with previously reported literature values^6,18^. Furthermore, they are independent of the cohesin under investigation and are unaffected by the presence or absence of Xyn-Doc124 during the measurement (see Table S1(a,c)). This confirms precise relative force comparability among the cohesins under investigation. All cohesins show their expected unfolding force distributions without Xyn-Doc124 in the measurement buffer, which we already determined in previous work^10^. As expected, all cohesin unfolding force distributions except those of Coh1 and T107S can be fitted adequately using the conventional two-state Bell-Evans model to obtain their most probable rupture forces (dashed lines)^21,22^. The rupture force histograms of Coh1 and T107S are more complex, indicating a multi-barrier unfolding energy landscape or multiple folds, and were therefore processed with kernel density estimation (KDE) to obtain most probable rupture forces from them (dotted lines). This atypical unfolding behavior might be explained by a distinctly flexible region critical for cohesin stability, for a detailed discussion see our previous work^10^. Since mutant T107S exhibits a bimodal unfolding force distribution, two most probable rupture forces F_1_ and F_2_ were extracted.

Upon addition of Xyn-Doc124 to the measurement buffer, all cohesins unfolded at notably higher forces (see Table S1(b)) and now all cohesin unfolding force distributions, including Coh1’s and T107S’, could be fitted with the Bell-Evans model (solid lines). Coh4 shows the smallest change in most probable rupture force with an increase of 31 pN (+6%). Cohesins number 2, 3, 6, 7 and mutant GGS all show an increase of 40 pN (+11%) to 84 pN (+17%). Interestingly, both Coh1 and mutant T107S, which displayed an atypical unfolding force distribution without dockerin, exhibit the largest increase in unfolding force upon addition of the dockerin, with ∆F_Coh1_ = 154 pN (+116%), and ∆F_T107S,1_ = 235 pN (+222%) and ∆F_T107S,2_ = 73 pN (+27%). This remarkable mechanical stabilization and the recovery of the Bell-Evans shape in their unfolding force distributions hint that the folds of Coh1 and T107S stabilize upon binding of the dockerin and that unfolding is now dominated by a single barrier. The average increase in most probable unfolding force of all cohesins under investigation at a loading rate which corresponds to an AFM pulling speed of 1600 nm/s was 95 pN (+27%), while both the ddFLN4 fingerprint unfolding and the CohIII-XDocIII unbinding showed no notable change (see Table S1).

### Negative control

To rule out a stabilizing effect caused by the Xyn domain or any other unknown effect caused by the addition of 5 µM protein on the cohesins’ unfolding behaviour, we added 5 µM xylanase-dockerinS (Xyn-DocS) instead of Xyn-Doc124 in a second measurement. This measurement served as a negative control, since DocS from *C. thermocellum’s* exocellulase Cel48S is known to not bind any of the cohesins under investigation^17^. We tested the change in unfolding force of the four cohesins which showed the strongest change under addition of Xyn-Doc124 (Coh1, Coh3, Coh5 and T107S) and found no notable change in unfolding behaviour for any of them (see Figure S2 and Table S3). This result confirms that the increase in mechanical stability upon addition of Xyn-Doc124 is in fact induced by the specific binding of Doc124 to ScaA’s cohesins, and not the presence of Xyn.

### Characterization of the free-energy landscape of Coh1 and Coh5

To better characterize the underlying conformational changes resulting in the increased mechanostability of the cohesins upon binding of the dockerins, we recorded dynamic force spectra of Coh1 and Coh5. The forces needed to unfold the two cohesins - without and with dockerin bound - were measured as a function of the loading rate by varying the AFM pulling speed between 400 nm/s, 800 nm/s, 1600 nm/s and 3200 nm/s.

**Figure 4:**
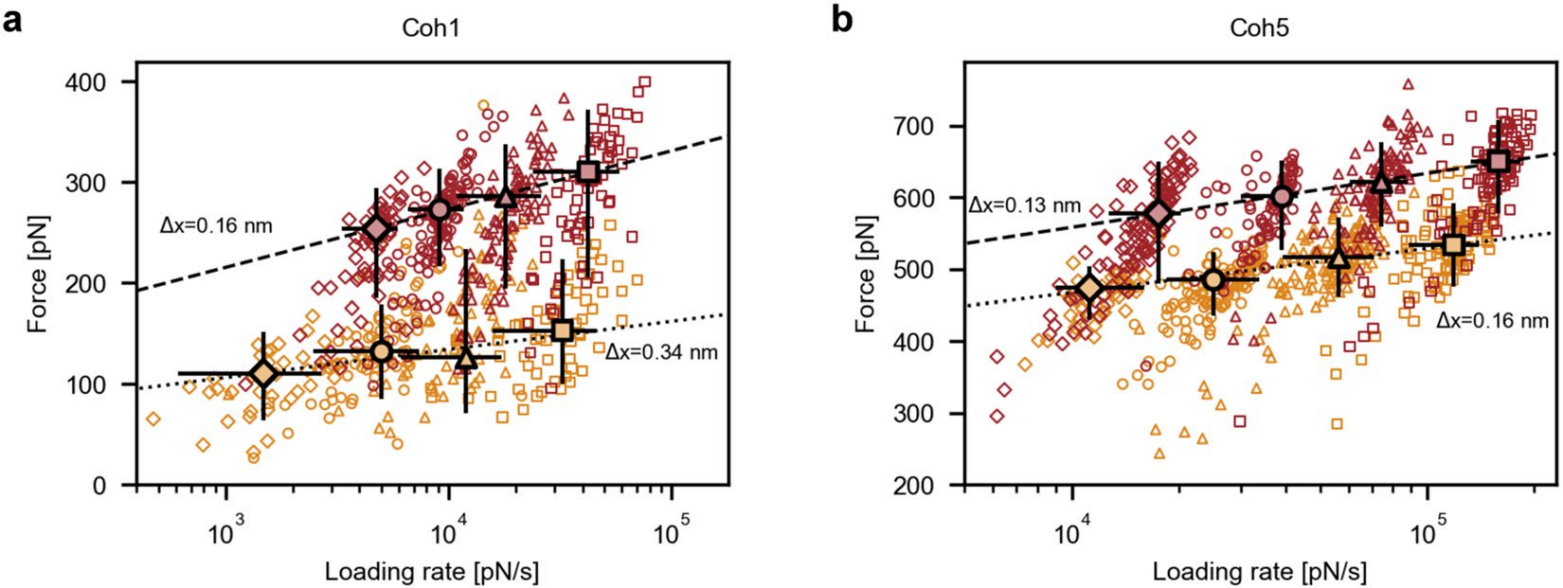
Dynamic force spectra for unfolding of Coh1 and Coh5, both without and with dockerin bound (orange and red, respectively). Diamonds, circles, triangles and squares represent pulling speeds of 400 nm/s, 800 nm/s, 1600 nm/s and 3200 nm/s, respectively. Large markers represent the most probable rupture force/loading rates of each speed. Error bars represent the full widths at half maxima. Fits of the Bell-Evans model through the most probable rupture force/loading rates of each speed are represented by dotted and broken lines (no dockerin and dockerin bound, respectively) (**a**) Fitting the Bell-Evans model to the most probable unfolding events of Coh1 of each pulling speed yields the distance to the transition state (±SD) of ∆x = 0.34 ± 0.11 nm and zero-force off rate k_0 _= (1.3 ± 4.1) 10^−2^ s^−1^ for the cohesin alone, and ∆x = 0.16 ± 0.01 nm and k_0 _= (8.6 ± 4.0) 10^−3^ s^−1^ with a dockerin bound. (**b**) The Bell-Evans fit to the most probable unfolding events of Coh5 of each pulling speed yields distances to the transition state (± SD) of ∆x = 0.16 ± 0.02 nm and zero-force off rate k_0 _= (11.7 ± 9.3) 10^−6^ s^−1^, for the cohesin alone, and ∆x = 0.13 ± 0.01 nm and k_0 _= (9.1 ± 2.1) 10^−6^ s^−1^, with a dockerin bound. All data were recorded using a single cantilever with a spring constant of 117 pN/nm during a 24 hour automated SMFS experiment.

We chose Coh1 because of its transition from an atypical unfolding force distribution to one which can be described by the Bell-Evans theory for single-barrier unfolding events. Additionally, Coh1 shows the largest absolute increase in most probable unfolding force of 154pN. Coh5 was chosen because it is mechanically the strongest of all cohesins under investigation, both with and without a dockerin bound, and because it exhibits the second largest absolute increase in unfolding force of 84 pN. Figure 4 shows the dynamic force spectra for Coh1 and Coh5, without and with dockerin bound, in dark and bright red, respectively. Although Coh1’s unfolding force distribution with no dockerin bound cannot be described by a Bell-Evans distribution, its dynamic force spectrum shows a linear dependence of the unfolding force on the logarithm of the force loading rate, which is predicted by the Bell-Evans model for dynamic force spectroscopy. We therefore chose to fit all dynamic force spectra, including Coh1’s, with the Bell-Evans model to obtain the parameters ∆x, the distance to transition state in the protein unfolding energy landscape, and k_0_, the zero-force off rate, and evaluate the change in the obtained kinetic parameters upon dockerin binding.

The fit of the Bell-Evans model to Coh1’s unfolding events without dockerin reveals an atypically large distance to the transition state (±SD) of ∆x = 0.34 ± 0.11 nm compared to previously measured values for cohesins of 0.11 nm to 0.17 nm^5,9,10^. The binding of the dockerin reduces this value to ∆x = 0.16 ± 0.01 nm. This is an indication that Coh1 undergoes a transition from one or more relatively weak fold conformations to a more stable folding state. The distance to the transition state of the strongest probed cohesin Coh5 slightly decreases from ∆x = 0.16 ± 0.02 nm to ∆x = 0.13 ± 0.01 nm, indicating a minor to no increase of the rigidity of its fold upon ligand binding.

## Conclusions

In summary, we characterized the change in mechanical stability of all seven cohesins from *A. cellulolyticus’* primary scaffoldin ScaA and two mutants of Coh1, utilizing a parallelized and high-throughput AFM-based SMFS protocol. All cohesins under investigation remarkably increased their mechanical stability upon binding of the dockerin, even though ligand binding takes place at the opposite site of the cohesins main structural element - i.e. the mechanical clamp motif - and the physical opening of the fold. While a recent study by Galera-Prat *et al*. found that two cohesins from *C. thermocellum’s* scaffoldin CipA did not change their mechanical resilience in the presence of a ligand^11^, we determined an average increase in most probable unfolding force for the cohesins of *A. cellulolyticus’* scaffoldin ScaA of 95 pN (+27%) upon dockerin binding at a loading rate which corresponds to an AFM pulling speed of 1600 nm/s. Additionally, the change in unfolding force distributions of Coh1 and T107S indicates that both cohesins were stabilized by dockerin binding. We hypothesize that Coh1 and T107S transition either from an ensemble of folding states or *via* several unfolding pathways to the unfolded state, unless stabilized in a single fold through binding of the dockerin, resulting in an unfolding behaviour dominated by a single barrier. This hypothesis is further strengthened by the observation that Coh1’s distance to the transition state decreases when its ligand is bound. Moreover, this suggests that Coh1 transitions through several fold conformations - similar in fold energy and separated only by minor barriers - during unfolding in the absence of dockerin.

We have employed diverse experimental techniques in this study, including the fast and parallelized one-pot *in vitro* protein expression and site-specific pulldown, highly specific pulling handles and molecular fingerprints, and parallel measurement of a protein library with a single cantilever. The combination of these experimental protocols allowed us to characterize the mechanical stability of nine receptors - with and without a ligand bound - within one 24-hour AFM-based SMFS experiment. Combined with recent developments in AFM-based SMFS, which have both accelerated data acquisition and improved data quality, the allosteric effects on protein mechanics induced by receptor-ligand binding can be screened more rapidly and accurately^15,16,23–26^.

## Materials and Methods

All reagents were at least of analytical purity grade and all buffers were filtered using a 0.2 µm polyethersulfone membrane filter prior to use. All incubation steps were done at room temperature, if not otherwise stated. All protocols follow Verdorfer *et al*.^10^. All data and constructs are available upon reasonable request.

### Gene construction, protein expression and purification

All genes were codon optimized for *E. coli*, synthesized and cloned into pET28a vectors. Plasmid DNA for cohesins mutants T107S and GGS was constructed from Coh1’s plasmid DNA using individually designed primers. All plasmids used in *in vitro* protein expression were amplified in DH5-alpha cells, purified using the QIAprep Spin Miniprep Kit (Qiagen, Hilden, Germany), eluted with ultrapure water and stored at −20 °C. All sequences were finally checked by DNA sequencing (Eurofins Genomics GmbH, Ebersberg, Germany).

The pulling handle and fingerprint protein CohIII-ddFLN4-HIS-ybbR, as well as both Xylanase-Dockerin (xylanase is typically used to enhance dockerin expression and folding^27^) constructs were expressed in *E. coli* NiCo21(DE3) cells (New England Biolabs, MA, USA) and purified using a Ni-NTA column for HIS-Tag purification. Both dockerins (Doc124 and DocS) could not to be expressed with their natural cellulases attached. The proteins were concentrated and exchanged into measurement buffer (TBS - Ca: 25 mM Tris, 72 mM NaCl, 1 mM CaCl2, pH 7.2) using desalting columns. They were frozen with 25% v/v glycerol in liquid nitrogen to be stored at −80 °C until used in experiments. All protein sequences can be found in the SI.

### AFM Sample preparation

Both the AFM cantilevers and the microscope slides were silanized using (3-aminopropyl)-dimethyl-ethoxysilane. A multiwell mask was attached to the glass slide to allow compartmentalization of the surface in spots with a diameter of ~500 µm. Both the cantilevers and the individual surfaces in the wells were incubated with 20 mM NHS-PEG-Maleimide (5 kDa) in 100 mM HEPES buffer pH 7.4 for 45 minutes. After rinsing with ultrapure water, both the cantilevers and the surfaces were incubated with 1 mM Coenzyme A (CoA), which bonds to the PEG’s maleimide group, in a 1 mM sodium phosphate pH 7.2, 50 mM NaCl, 10 mM EDTA buffer for 2 hours. After a final ultrapure water rinse the cantilevers were incubated with 40 μM Coh3-ddFLN4-HIS-ybbR and 5 μM phosphopantetheinyl transferase (Sfp)^28^ for 2 hours in magnesium chloride supplemented measurement buffer (TBS- Ca: 25 mM Tris, 72 mM NaCl, 1 mM CaCl2, 20 mM MgCl_2_ pH 7.2). The Sfp covalently bonds the Coh3-ddFLN4-HIS-ybbR to the PEG-CoA linkers on the cantilever. The cantilevers were rinsed extensively with measurement buffer and finally stored in it until use in measurement.

### *In vitro* expression and protein pulldown

The IVTT reaction mixes consisting of the PURExpress® kit, 5 μM Sfp^28^, 0.8 U/µl RNase inhibitor, 10 ng/μl Plasmid-DNA (encoding for ybbR-Coh-XDocIII) and 0.05% v/v Triton X-100 were transferred to the wells and incubated at 37 °C for 3 h. The proteins were both expressed, and bound to the PEG-CoA linkers on the surface by the Sfp *via* their ybbR-tag during this time. The individual wells were finally rinsed extensively using measurement buffer and the multiwell mask was removed.

### AFM SMFS measurements

A custom-build AFM, specialized for multispot SMFS, was used for all measurements. The cantilever was aligned to all individual protein spots and the positions were stored in the control software. All single-speed SMFS measurements were done with at a constant pulling velocity of 1600 nm/s. The dynamic force spectra were recorded at a pulling speed of 400 nm/s, 800 nm/s, 1600 nm/s, and 3200 nm/s. After 2000 approach- and retract-cycles in one protein spot the AFM head was automatically moved to the next one. The measurement buffer on the sample was exchanged after 6 to 12 hours with measurement buffer containing 5 µM of Xylanase-Dockerin; Doc124 from *A. cellulolyticus* or DocS (taken from exocellulase Cel48S) from *C. thermocellum* for main measurement and dynamic force spectra or negative control, respectively.

### AFM SMFS data Analysis

Data analysis was carried out following previous work^10,19^. Laser spot drift on the cantilever relative to the calibration curve can be significant in 24-hour long measurement sessions. It was therefore corrected via the baseline noise (determined as the last 5% of data points for each curve) for all curves and smoothed with a moving median. The inverse optical lever sensitivity (InvOLS) for each curve was linearly corrected relative to the InvOLS value of the calibration curve according to the baseline noise. All curves showing a ddFLN4 and cohesin contour length increment (ddFLN4: 34 nm^18,19^, cohesin: 45 nm) were used to assemble unfolding force histograms, which were then fitted either following the Bell-Evans model^22,29^ or using kernel density estimates. Both methods were used to obtain most probable rupture forces.

Bell-Evans probability density function at given loading rate r with fit parameters distance to the transition state ∆x and natural off-rate k_0_:

$$p(F)=\frac{{k}_{0}}{r}\,{\exp }[\frac{\Delta x}{{k}_{B}T}F-\frac{{k}_{0}\,{k}_{B}T}{r\,\Delta x}({e}^{\frac{\Delta x}{{k}_{B}T}F}-1)]$$

The Bell-Evans model predicts a linear dependence between the most probable rupture force <F> and the logarithm of the force loading rate r in dynamic force spectra:

$$ < \,F(r)\, > =(\frac{{k}_{B}T}{\Delta x})\,ln\,(\frac{r\Delta x}{{k}_{0}{k}_{B}T})$$

The standard deviation for the fitted parameters Δx and k_0_ were obtained by taking the square root of the diagonal entries of the covariance matrix from the fitting algorithm. We used the Levenberg-Marquardt algorithm from the Scipy python library^30,31^.

## Electronic supplementary material

Supplementary information
