# Supplementary material for: Ligand Binding Stabilizes Cellulosomal Cohesins as Revealed by AFM-based Single-Molecule Force Spectroscopy
Source: Sci Rep. 2018 Jun 25;8:9634. doi: 10.1038/s41598-018-27085-x (PMC6018229; doi:10.1038/s41598-018-27085-x)
Supplement: Supplementary file 1 — Supplementary information [file 41598_2018_27085_MOESM1_ESM.pdf]

Supporting Information for

# Ligand Binding Stabilizes Cellulosomal Cohesins as Revealed by AFM-based Single-Molecule Force Spectroscopy

Tobias Verdorfer*,[1] and Hermann E. Gaub[1]

[1] Lehrstuhl für Angewandte Physik and Center for Nanoscience, Ludwig-Maximilians-Universität, 80799 Munich, Germany.

*Correspondence and requests for materials should be addressed to TV (email: tobias.verdorfer@physik.uni-muenchen.de).

KEYWORDS Single molecule force spectroscopy, AFM, cohesin-dockerin, cellulosome, mechanical stability, receptor-ligand, allostery

(a) ddFLN4 unfolding forces F [pN]

| | most probable | | | | mean | | | |
|---|---|---|---|---|---|---|---|---|
| | no doc | doc | ΔF | | no doc | doc | ΔF | |
| Cohesin 1 | 82 | 81 | -2 | -2.1% | 80 | 76 | -4 | -5.3% |
| Cohesin 2 | 86 | 82 | -4 | -4.9% | 82 | 79 | -2 | -3.0% |
| Cohesin 3 | 82 | 78 | -3 | -4.1% | 78 | 77 | -1 | -1.8% |
| Cohesin 4 | 81 | 78 | -3 | -4.0% | 79 | 75 | -4 | -5.0% |
| Cohesin 5 | 81 | 78 | -2 | -3.1% | 78 | 83 | 5 | 6.4% |
| Cohesin 6 | 82 | 78 | -4 | -4.9% | 82 | 78 | -3 | -3.9% |
| Cohesin 7 | 77 | 77 | 0 | -0.3% | 79 | 75 | -3 | -4.1% |
| Cohesin T107S | 77 | 82 | 5 | 6.1% | 80 | 79 | -2 | -2.0% |
| Cohesin GGS | 77 | 81 | 5 | 6.1% | 79 | 77 | -2 | -2.8% |
| **average** | **81** | **79** | **-1** | **-1.4%** | **80** | **78** | **-2** | **-2.4%** |

(d) Number of curves

| | no doc | doc |
|---|---|---|
| Cohesin 1 | 149 | 266 |
| Cohesin 2 | 191 | 274 |
| Cohesin 3 | 201 | 258 |
| Cohesin 4 | 170 | 256 |
| Cohesin 5 | 154 | 167 |
| Cohesin 6 | 191 | 222 |
| Cohesin 7 | 210 | 219 |
| Cohesin T107S | 192 | 272 |
| Cohesin GGS | 72 | 129 |
| sum | 1530 | 2063 |

(b) Cohesin unfolding forces F [pN]

| | most probable | | | | mean | | | |
|---|---|---|---|---|---|---|---|---|
| | no doc | doc | ΔF | | no doc | doc | ΔF | |
| Cohesin 1 | 134 | 288 | 154 | **115.6%** | 153 | 262 | 109 | **71.5%** |
| Cohesin 2 | 362 | 415 | 53 | **14.8%** | 336 | 395 | 59 | **17.5%** |
| Cohesin 3 | 315 | 369 | 54 | **17.2%** | 291 | 350 | 59 | **20.1%** |
| Cohesin 4 | 510 | 541 | 32 | **6.2%** | 478 | 522 | 43 | **9.1%** |
| Cohesin 5 | 525 | 609 | 84 | **16.1%** | 474 | 584 | 109 | **23.0%** |
| Cohesin 6 | 418 | 465 | 48 | **11.4%** | 387 | 455 | 68 | **17.7%** |
| Cohesin 7 | 465 | 529 | 64 | **13.7%** | 440 | 512 | 72 | **16.3%** |
| Cohesin T107S | 106 / 268 | 341 | 235 / 73 | **221.7% / 27.1%** | 226 | 319 | 93 | **41.0%** |
| Cohesin GGS | 373 | 414 | 40 | **10.7%** | 350 | 391 | 41 | **11.6%** |
| **average** | **348** | **441** | **94** | **27.0%** | **348** | **421** | **72** | **20.8%** |

(e)

| | |
|---|---|
| lever spring constant: | 143pN/nm |
| time w/o doc: | ~12hrs |
| time w/ doc: | ~12hrs |
| total exp time: | ~24hrs |

(c) CohIII:XDocIII unbinding forces F [pN]

| | most probable | | | | mean | | | |
|---|---|---|---|---|---|---|---|---|
| | no doc | doc | ΔF | | no doc | doc | ΔF | |
| Cohesin 1 | 693 | 674 | -18 | -2.6% | 675 | 659 | -16 | -2.3% |
| Cohesin 2 | 693 | 700 | 8 | 1.1% | 666 | 669 | 2 | 0.4% |
| Cohesin 3 | 702 | 694 | -8 | -1.2% | 677 | 666 | -11 | -1.6% |
| Cohesin 4 | 685 | 701 | 16 | 2.3% | 669 | 674 | 5 | 0.8% |
| Cohesin 5 | 703 | 700 | -3 | -0.5% | 680 | 669 | -11 | -1.6% |
| Cohesin 6 | 715 | 710 | -4 | -0.6% | 670 | 665 | -4 | -0.7% |
| Cohesin 7 | 698 | 691 | -7 | -1.0% | 678 | 658 | -20 | -3.0% |
| Cohesin T107S | 700 | 702 | 2 | 0.3% | 665 | 665 | -1 | -0.1% |
| Cohesin GGS | 683 | 727 | 44 | 6.4% | 667 | 676 | 9 | 1.3% |
| **average** | **697** | **700** | **3** | **0.5%** | **672** | **667** | **-5** | **-0.8%** |

Table S1: Summary of all unfolding force data from main experiment. (a)-(c) Most probable and mean rupture for the ddFLN4 fingerprint, all cohesins of interest and the CohIII-XDocIII pulling handle. Neither the ddFLN4's nor the CohIII-XDocIII's most probable or mean unfolding forces show a notable change upon addition of the dockerin Doc124, while all cohesins show a considerable increase. (d) Number of force curves of all molecular constructs. (e) General experiment information.

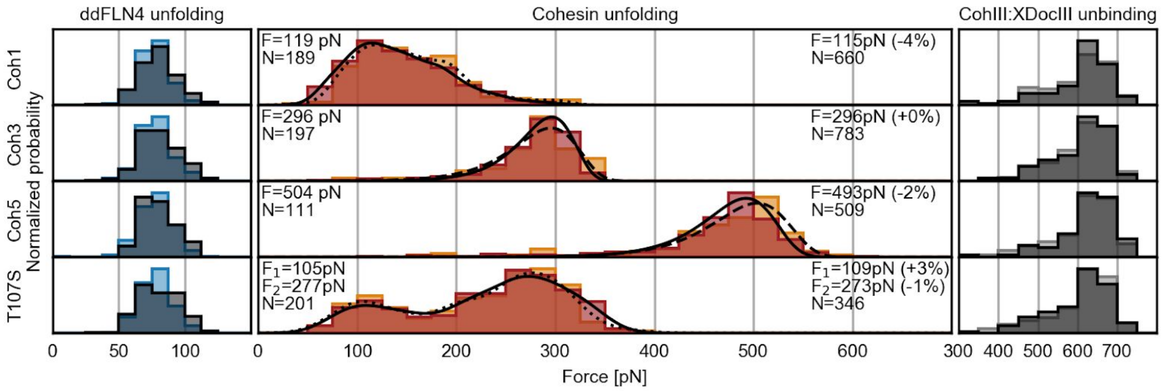


Figure S2: Negative control: Unfolding and rupture force histograms without and with Xyn-DocS in solution. Tested were the four cohesins with the strongest change unfolding force under addition of Xyn-Doc124: Coh1, Coh3, Coh5, and T107S. Both the unfolding force histograms of the fingerprint (left column, bright and dark blue without and with dockerin in solution, respectively) and the unbinding force histograms of the CohIII-XDocIII pulling handle (right column, bright and dark grey without and with dockerin in solution, respectively) are independent of the cohesin under investigation and unaffected of whether or not Xyn-DocS is present during measurement. All cohesins under investigation show their expected and previously known unfolding force distributions without Xyn-DocS in solution[4] (middle column, orange). As expected, Coh3 and Coh5 can be fitted using the Bell-Evans model to obtain their most probable unfolding forces (dashed lines). The rupture force histograms of Coh1 and T107S were smoothed using kernel density estimations (KDE) to obtain most probable unfolding forces from them (dotted lines). The unfolding force distributions, and thus the Bell-Evans fits and KDEs, of all cohesins remain virtually unchanged upon the addition of Xyn-DocS (middle column, red, solid lines). Forces F on the left and right side of the middle column represent the most probable rupture forces of the cohesins without and with Xyn-DocS in solution, respectively, and their relative change in percent. Numbers N on the left and on the right side of the middle column represent the number of cohesin unfolding force data points in the histograms without and with dockerin in solution, respectively. We found that changing the bin size within a range of 10pN to 50pN did not notably alter the fits of the Bell-Evans model to the cohesin unfolding force histograms or the obtained most probable unfolding forces. We therefore chose the same bin size for all constructs within a column of histograms (25pN for all ddFLN4 and cohesin unfolding events, and 50pN for all CohIII-XDocIII unbinding events), to provide good comparability by eye. All data were recorded using a single cantilever with a spring constant of 114pN/nm at a retraction speed of 1600 nm/s in a 24 hour automated SMFS experiment.

(a)

**ddFLN4 unfolding forces F [pN]**

| | most probable | | | | mean | | | |
|---|---|---|---|---|---|---|---|---|
| | no doc | doc | ΔF | | no doc | doc | ΔF | |
| Cohesin 1 | 78 | 78 | 0 | 0.0% | 80 | 77 | -3 | -4.0% |
| Cohesin 3 | 76 | 79 | 3 | 4.1% | 80 | 78 | -2 | -3.0% |
| Cohesin 5 | 75 | 76 | 1 | 1.1% | 80 | 78 | -2 | -2.6% |
| Cohesin T107S | 73 | 79 | 5 | 7.0% | 80 | 78 | -2 | -2.3% |
| **average** | **76** | **78** | **2** | **3.0%** | **80** | **78** | **-2** | **-3.0%** |

(b)

**Cohesin unfolding forces F [pN]**

| | most probable | | | | mean | | | |
|---|---|---|---|---|---|---|---|---|
| | no doc | doc | ΔF | | no doc | doc | ΔF | |
| Cohesin 1 | 119 | 115 | -4 | **-3.6%** | 151 | 143 | -8 | **-5.2%** |
| Cohesin 3 | 296 | 296 | 1 | **0.2%** | 277 | 277 | 0 | **-0.1%** |
| Cohesin 5 | 504 | 493 | -11 | **-2.2%** | 468 | 465 | -3 | **-0.7%** |
| Cohesin T107S* | 105 277 | 109 273 | 3 -4 | **2.9% -1.4%** | 223 | 229 | 6 | **2.7%** |
| **average** | **260** | **257** | **-3** | **-1.2%** | **280** | **278** | **-1** | **-0.5%** |

(c)

**CohIII:XDocIII unbinding forces F [pN]**

| | most probable | | | | mean | | | |
|---|---|---|---|---|---|---|---|---|
| | no doc | doc | ΔF | | no doc | doc | ΔF | |
| Cohesin 1 | 632 | 645 | 13 | 2.1% | 604 | 604 | 1 | 0.1% |
| Cohesin 3 | 645 | 643 | -2 | -0.4% | 605 | 607 | 2 | 0.3% |
| Cohesin 5 | 648 | 650 | 2 | 0.2% | 624 | 615 | -10 | -1.6% |
| Cohesin T107S | 637 | 646 | 9 | 1.4% | 602 | 606 | 4 | 0.6% |
| **average** | **640** | **646** | **5** | **0.8%** | **609** | **608** | **-1** | **-0.1%** |

(d)

**Number of curves**

| | no doc | doc |
|---|---|---|
| Cohesin 1 | 189 | 660 |
| Cohesin 3 | 197 | 783 |
| Cohesin 5 | 111 | 509 |
| Cohesin T107S | 201 | 346 |
| sum | 698 | 2298 |

(e)

| | |
|---|---|
| lever spring constant: | 114pN/nm |
| time w/o doc: | ~6hrs |
| time w/ doc: | ~18hrs |
| total exp time: | ~24hrs |

Table S3: Summary of all unfolding force data from negative control experiment. (a)-(c) Most probable and mean rupture for the ddFLN4 fingerprint, all cohesins of interest and the CohIII-XDocIII pulling handle. None of the molecular constructs show a notable change in most probable or mean unfolding forces upon addition of the dockerin DocS. (d) Number of force curves of all molecular constructs. (e) General experiment information.

# Protein sequences

ybbR-tag - linker and additional residues - XylanaseT6 (B. stea) - linker - Doc124 (*A. cellulolyticus*):

MGTDSLEFIASKLALEVLFQGPLQHHHHHHPWTSASKNADSYAKKPHISALNAPQLDQRYKNEFTIGAAVEPYQLQNEKDVQML
KRHFNSIVAENVMKPISIQPEEGKFNFEQADRIVKFAKANGMDIRFHTLVWHSQVPQWFFLDKEGKPMVNECDPVKREQNKQLLL
KRLETHIKTIVERYKDDIKYWDVVNEVVGDDGKLRNSPWYQIAGIDYIKVAFQAARKYGGDNIKLYMNDYNTEVEPKRTALYNL
VKQLKEEGVPIDGIGHQSHIQIGWPSEAEIEKTINMFAALGLDNQITELDVSMYGWPPRAYPTYDAIPKQKFLDQAARYDRLFKLYE
KLSDKISNVTFWGIADNHTWLDSRADVYYDANGNVVVDPNAPYAKVEKGKGKDAPFVFGPDYKVKPAYWAIIDHKVVPAVTGD
INGDGYFNSIDFGLMRVYLLSGSIPNYSAADVNGDSNANSIDFGYMRQYLLGIITVFPNGGTQT

ybbR-tag - linker and additional residues - XylanaseT6 (B. stea) - linker - DocS (*C. thermocellum*):

MGTDSLEFIASKLALEVLFQGPLQHHHHHHPWTSASKNADSYAKKPHISALNAPQLDQRYKNEFTIGAAVEPYQLQNEKDVQML
KRHFNSIVAENVMKPISIQPEEGKFNFEQADRIVKFAKANGMDIRFHTLVWHSQVPQWFFLDKEGKPMVNECDPVKREQNKQLLL
KRLETHIKTIVERYKDDIKYWDVVNEVVGDDGKLRNSPWYQIAGIDYIKVAFQAARKYGGDNIKLYMNDYNTEVEPKRTALYNL
VKQLKEEGVPIDGIGHQSHIQIGWPSEAEIEKTINMFAALGLDNQITELDVSMYGWPPRAYPTYDAIPKQKFLDQAARYDRLFKLYE
KLSDKISNVTFWGIADNHTWLDSRADVYYDANGNVVVDPNAPYAKVEKGKGKDAPFVFGPDYKVKPAYWAIIDHKVVPGTPST
KLYGDVNDDGKVNSTDAVALKRYVLRSGIGSGSGSGSGSGSGSGSSINTDNADLNEDGRVNSTDLGILKRYILKEIDTLPYKN

ybbR-tag - linker and additional residues - Cohesin - linker - XDoc3:

MGTDSLEFIASKLALEVLFQGPLQHHHHHHPWTSAS

Cohesin 1
TGFTVNVDSVNGNVGEQIVVPVSFANVPSNGVSTADMTITYDSSKLEYVSGAAGSIVTNPTVNFGINKEADGKLKVLFLDYTMSTG
YISTNGVFANVTFKVLNSAPTTVGITGATFGDKNLGNISATINAGSINGG

Cohesin 2
TGFTVNVDSVNGNVGEQIVVPVSFANVPSNGISTADMTITYDSSKLEYVSGDAGSIVTNPTVNFGINKETDGKLKVLFLDYTMSTG
YISTNGVFAKVTFKVLNAGGSSVGITGATFGDKNLGSVSATINAGSINGG

Cohesin 3
TGFTVSVDSVNGNVGEQIVIPVSFANIPANGISTADMTITYDSSKLEYVSGVPGSIVTNPDVNFGINKETDGKLKVLFLDYTMSTGYI
STSGVFTKVTFKVLSSGGSTVGITGATFGDKNLGNVSATINAGSINGG

Cohesin 4
NAMAVAVGAVQGGVGETVTVPVTMTKVPTTGVSTADFTVTYDATKLEYVSGAAGSIVTNPDVNFGINKEADGKIKVLFLDYTM
ATEYISKDGVFANLTFKIKSTAAAGTTAAVGIAGTATFGDSALKPITAVITDGKVEII

Cohesin 5
KAMKVVIANVSGNAGSEVVVPVSIEGVSANGVSAADFTITYDATKLDYVSGAAGSIVKNPDVNFGINKEADGKLKVLFLDYTMAT
EYISADGIFANLTFKIKSTAVNGDVAAISKSGTATFGDKNLGPISAVIKDGSVTVG

Cohesin 6
TGFNLSIDTVEGNPGSSVVVPVKLSGISKNGISTADFTVTYDATKLEYISGDAGSIVTNPGVNFGINKESDGKLKVLFLDYTMSTGYI
STDGVFANLNFNIKSSAAIGSKAEVSISGTPTFGDSTLTPVVAKVTNGAVNVV

Cohesin 7
NAFKVSIDTVKAATGTQVVVPVSFVNVPATGISTTDMTITYDATKLQYVSGDAGSIVTNPGVNFGINKEADGKLKVLFLDYTMTT
QYISEDGVFANVTFKVIGTDGLAAVNAEDATFGDSSLSPVTASVVNGGVNIG

Cohesin 1 A105G P106G T107S "GGS"
TGFTVNVDSVNGNVGEQIVVPVSFANVPSNGVSTADMTITYDSSKLEYVSGAAGSIVTNPTVNFGINKEADGKLKVLFLDYTMSTG
YISTNGVFANVTFKVLNS**GGS**TVGITGATFGDKNLGNISATINAGSINGG

Cohesin 1 T107S
TGFTVNVDSVNGNVGEQIVVPVSFANVPSNGVSTADMTITYDSSKLEYVSGAAGSIVTNPTVNFGINKEADGKLKVLFLDYTMSTG
YISTNGVFANVTFKVLNSAP**S**TVGITGATFGDKNLGNISATINAGSINGG

VVPNTVTSAVKTQYVEIESVDGFYFNTEDKFDTAQIKKAVLHTVYNEGYTGDDGVAVVLREYESEPVDITAELTFGDATPANTYK
AVENKFDYEIPVYYNNATLKDAEGNDATVTVYIGLKGDTDLNNIVDGRDATATLTYYAATSTDGKDATTVALSPSTLVGGNPESV
YDDFSAFLSDVKVDAGKELTRFAKKAERLIDGRDASSILTFYTKSSVDQYKDMAANEPNKLWDIVTGDAEEE

Coh3 - linker - ddFLN4 - linker and additional residues - ybbR

MGTALTDRGMTYDLDPKDGSSAATKPVLEVTKKVFDTAADAAGQTVTVEFKVSGAEGKYATTGYHIYWDERLEVVATKTGAY
AKKGAALEDSSLAKAENNGNGVFVASGADDDFGADGVMWTVELKVPADAKAGDVYPIDVAYQWDPSKGDLFTDNKDSAQGK
LMQAYFFTQGIKSSSNPSTDEYLVKANATYADGYIAIKAGEPGSVVPSTGSADPEKSYAEGPGLDGGESFQPSKFKIHAVDPDGVH
RTDGGDGFVVTIEGPAPVDPVMVDNGDGTYDVEFEPKEAGDYVINLTLDGDNVNGFPKTVTVKPAPGSELKLPRSRHHHHHHGS
LEVLFQGPDSLEFIASKLA
